# Preliminary Evidence That High-Dose Vitamin C has a Vascular Disrupting Action in Mice

**DOI:** 10.3389/fonc.2014.00310

**Published:** 2014-11-05

**Authors:** Bruce C. Baguley, Qi Ding, Emma Richardson

**Affiliations:** ^1^Auckland Cancer Society Research Centre, Faculty of Medical and Health Sciences, The University of Auckland, Auckland, New Zealand

**Keywords:** antivascular, endothelial, tumor, vadimezan, fosbretabulin, serotonin, biomarker

## Abstract

High intravenous doses of vitamin C (ascorbic acid) have been reported to benefit cancer patients, but the data are controversial and there is incomplete knowledge of what physiological mechanisms might be involved in any response. Vitamin C is taken up efficiently by cells expressing SVCT2 transporters and since vascular endothelial cells express SVCT2, we explored the hypothesis that administration of high-dose vitamin C (up to 5 g/kg) to mice might affect vascular endothelial function. A single administration of vitamin C to mice induced time- and dose-dependent increases in plasma concentrations of the serotonin metabolite 5-hydroxyindole acetic acid (5-HIAA), a marker for vascular disrupting effects. Responses were comparable to those for the tumor vascular disrupting agents, vadimezan and fosbretabulin. High-dose vitamin C administration decreased tumor serotonin concentrations, consistent with the release of serotonin from platelets and its metabolism to 5-HIAA. High-dose vitamin C also significantly increased the degree of hemorrhagic necrosis in tumors removed after 24 h, and significantly decreased tumor volume after 2 days. However, the effect on tumor growth was temporary. The results support the concept that vitamin C at high dose increases endothelial permeability, allowing platelets to escape and release serotonin. Plasma 5-HIAA concentrations could provide a pharmacodynamic biomarker for vitamin C effects in clinical studies.

## Introduction

The physiological effects of orally administered vitamin C as an antioxidant and in preventing scurvy are well known (1–3). Vitamin C at physiological concentrations generally has a protective effect on the vascular endothelium, decreasing its permeability (3). Plasma concentrations of vitamin C in humans following high oral doses are limited by absorption, but intravenous administration can overcome this limitation and lead to millimolar plasma concentrations (4, 5). Vitamin C at high dose can inhibit tumor growth in mice (6–9) and early studies reported that administration of vitamin C at high intravenous doses to cancer patients increased survival by more than 5 months (10, 11). However, subsequent clinical studies were conflicting and there is currently no clear resolution of the question of whether high-dose intravenous vitamin C affects patient survival (12). Progress in our understanding of the action of high-dose vitamin C has been hampered by a lack of appropriate biomarkers for physiological responses to vitamin C.

There is good evidence that tumors have a disorganized and inefficient blood supply as compared to normal tissues. This leads to metabolic and hypoxic stress, to the induction of hypoxia-inducible factor-1α (HIF-1α) (13), and to increased production of vascular endothelial growth factor (VEGF), also called vascular permeability factor (14). The resulting increase in vascular permeability leads to reduced tumor blood flow (15). Vascular disrupting agents are thought to act by further increasing vascular permeability, thus reducing tumor blood flow to the point of vascular failure (16, 17). Several vascular disrupting agents have advanced to clinical trial, most notably vadimezan (DMXAA) in non-small cell lung cancer (18) and fosbretabulin (combretastatin A4-phosphate; CA4-P) in thyroid cancer (19). In this preliminary study, we have investigated the hypothesis that vitamin C administered at high dose can act as vascular disrupting agent. The basis for this hypothesis is that vascular endothelial cells, through expression of the SVCT2 transporter (2, 20, 21), concentrate cytoplasmic vitamin C to a point where it affects vascular function.

Measurement of the clinical effects of vascular disrupting agents has generally been carried out with the use of contrast-enhanced magnetic resonance (22, 23). A paramagnetic contrast agent such as gadolinium–diethylenetriamine pentaacetic acid, which binds tightly to albumin, is administered. Increased vascular permeability in tumor tissue leads to extravasation and tissue retention of this protein-bound agent, concentrations of which can be measured using magnetic resonance. In experimental studies, a similar strategy is employed using the dye Evans Blue, which like the gadolinium derivative is tightly bound to albumin in plasma. Increased vascular permeability in tumor tissue leads to extravasation of the albumin–dye complex, which can be measured by a photometric assay (24). In previous studies, we have proposed that extravasation of platelets could also be used to detect increased vascular permeability (16). Serotonin (5-hydroxytryptamine; 5-HT) is localized in platelets as a consequence of the action of vesicular monoamine transporters, which also concentrate serotonin into chromaffin granules of the adrenal medulla and into neurons (25). Tumor tissue concentrations of serotonin therefore largely reflect platelet content of serotonin. Increased vascular permeability leads to extravasation of platelets, to platelet activation (for instance, by extravascular contact with collagen) and to serotonin release (26). Free serotonin is converted by hepatic metabolism to 5-hydroxyindole acetic acid (5-HIAA), which can be measured by high-performance liquid chromatography (27, 28). Thus, increased vascular permeability leads to serotonin loss from platelet and to increased free concentrations of serotonin and 5-HIAA.

Previous preclinical studies have shown increased plasma concentrations of 5-HIAA and serotonin in response to a number of vascular disrupting agents including flavone acetic acid (FAA), vadimezan, vinblastine, and colchicine (27, 30, 31). In the case of vadimezan, increases in plasma 5-HIAA concentrations are highly correlated with increased extravasation of Evans Blue into tumor tissue as a marker of tumor vascular permeability (*r* = 0.82), as well as with decreased tumor blood flow (*r* = 0.73) (24, 32). A Phase 1 clinical trial of vadimezan has shown that plasma 5-HIAA concentrations increase in response to the drug (28, 29), and a Phase 2 trial has shown that increases in 5-HIAA correlate positively with dose (33). Here, we have measured increases in plasma 5-HIAA concentrations in mice treated with high doses of vitamin C, comparing them with those of vadimezan. We also include data showing the 5-HIAA response to the vascular disrupting agent fosbretabulin. We have examined the effect of high-dose vitamin C on tissue serotonin concentrations in tumor and liver, and on the growth of Colon 38 tumors in mice.

## Materials and Methods

### Materials

Vadimezan was synthesized in this laboratory (34), dissolved in sterile saline, protected from light, and injected into the peritoneal cavity in a volume of 10 μl/g body weight. Vitamin C was used as a solution in clinical vial (McGuff Pharmaceuticals Ltd., New Zealand) and diluted with water to the appropriate concentration before injection in a volume of 10 μl/g body weight. Fosbretabulin, kindly provided by Cancer Research UK, was dissolved in dimethylsulfoxide (10 mg/ml) and injected in a volume of 2.5 μl/g body weight. Other biochemicals were from Sigma.

### Mice and tumors

All experiments were approved by the Animal Ethics Committee of the University of Auckland. C57Bl/6 mice were bred and housed under controlled temperature, humidity, and lighting. Mice between 6 and 12 weeks of age (18–22 g) were used. Samples of murine Colon 38 carcinomas (1 mm^3^ tumor fragments from a carrier mouse) were implanted subcutaneously in mice that had been anesthetized by intraperitoneal administration (10 l/g body weight) of a xylazine (10 mg/kg)/ketamine (150 mg/kg) mixture. Treatment was commenced 12–14 days after implantation, when tumors were approximately 5 mm in diameter. Tumor growth delay experiments utilized groups of five mice; tumor size was measured with calipers and volumes were calculated as (*a*^2^*b*), where *a* and *b* are the minor and major tumor axes, respectively. Tumor histology was carried out on a group of mice with larger tumors (5–8 mm in diameter). Mice were treated with vitamin C (4 g/kg) and tumors were removed after 24 h, fixed with formalin, and sections stained with hematoxylin/eosin (35). The whole tumor section (largest diameter) was photographed and a grid was super-imposed on the photograph. Each section of the grid was then analyzed for hemorrhagic tumor necrosis (i.e., lack of stained nuclei) and the combined results used to derive an estimate of the proportion of necrosis.

### Measurement of serotonin and 5-HIAA in plasma and tumor samples

General methods for the measurement of plasma, tumor, and liver tissue concentrations of serotonin and 5-HIAA have been described previously (27, 36). For both the time course and dose dependence studies, blood was collected from groups of three terminally anesthetized mice through the ocular sinus into heparinized polypropylene microcentrifuge tubes containing 20 μl of 7.5% sodium ethylenediamine tetra-acetate (EDTA) and 1 mg sodium ascorbate. Tumor and liver tissue were homogenized in 7.5% EDTA/0.02% sodium ascorbate (1 ml for tumor tissue and 2 ml for in liver tissue). Tubes were centrifuged (5,000 × *g*; 10 min), and plasma samples were removed and deproteinized by addition of 1 ml of ice-cold acetonitrile:water (3:1) to 100 μl plasma. After mixing and centrifugation (3,000 × *g*; 15 min; 4°C), the supernatants were concentrated in a centrifugal vacuum concentrator, reconstituted in 100 μl mobile phase [80% acetonitrile:45 mM formate buffer (35:63)]. Liver and tumor tissue homogenates were resuspended in mobile phase.

5-HIAA was analyzed by high-performance liquid chromatography using a LUNA C18 5 μm 75 mm × 4.6 mm stainless steel column (Phenomenex, Torrence, CA, USA) with a Phenomenex C18 Security guard column. The mobile phase was 0.14 M potassium phosphate buffer pH 4.50 containing 15% methanol, 5% acetonitrile, and 0.004% acetyltrimethyl-ammonium bromide, 2 mM NaCl, and 0.1 mM EDTA. 5-HIAA was detected using a Model DECADE II Electrochemical Detector (Antec Leyden, Netherlands) with an electrode potential of 400 mV. Quantitation was carried out by reference to a standard curve. Serotonin was analyzed using a LUNA C8 5 μm 150 mm × 3.0 mm stainless steel with a Phenomenex C18 Security guard column. The mobile phase was 85% 0.14 M potassium phosphate buffer pH 4.50/15% acetonitrile together with 1 g/l 1-octansulfonic acid sodium salt. Serotonin was detected using a Shimadzu RF-10AXL fluorescence detector and quantitation utilized *N*-acetyl serotonin as an internal standard.

### Statistical analysis

Values are quoted as means ± standard errors. Groups were compared using routines available on SigmaPlot. Student’s *t*-test was used when the data were normally distributed and a Rank Sum test was used when the data were not normally distributed.

## Results

### Effects of vitamin C treatment on serotonin and 5-HIAA concentrations

**Figure 1:**
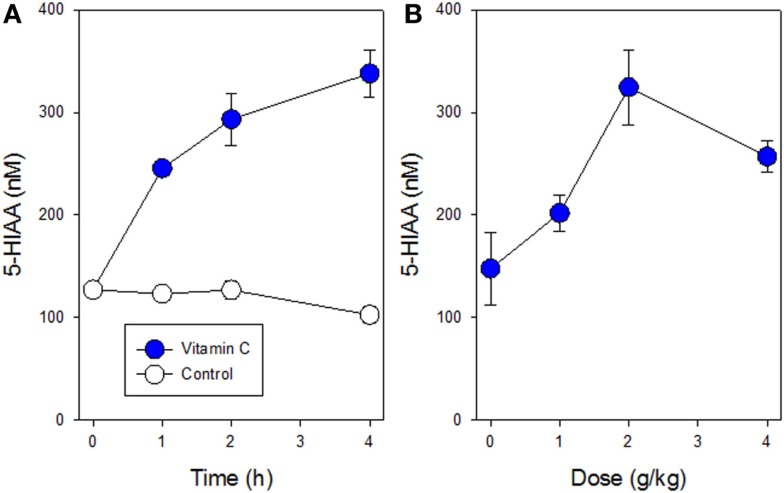
**Plasma concentrations of 5-HIAA following a single i.p. administration of vitamin C are shown**. **(A)** Time course for non-tumor-bearing mice following administration of vitamin C (5 g/kg; filled circles) or with no treatment (open circles). **(B)** Dose curve for non-tumor-bearing mice following administration of vitamin C (0–4 g/kg). Vertical bars in each case represent SEM.

**Figure 2:**
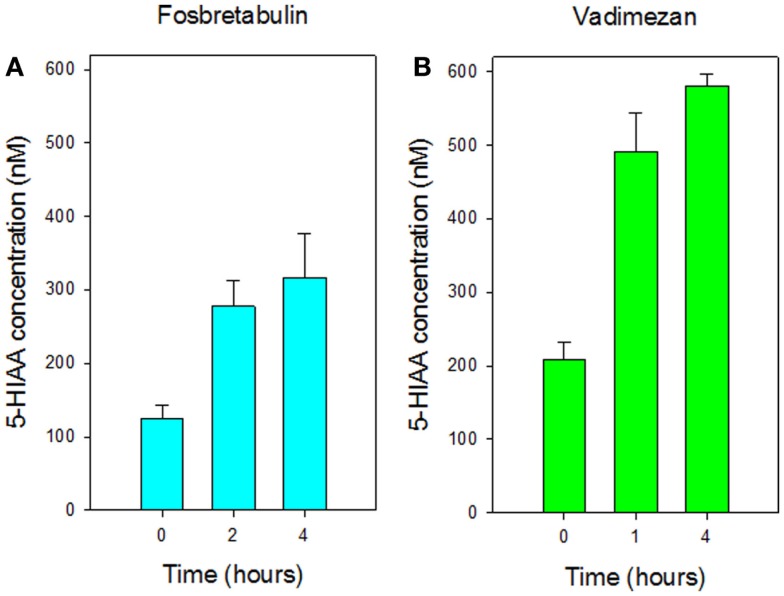
**Comparison of plasma concentrations of 5-HIAA in non-tumor-bearing mice following i.p. administration of (A) fosbretabulin (25 mg/kg) and (B) vadimezan (25 mg/kg)**. Data for vadimezan are extracted from a previous publication (36). Vertical bars represent SEM (three mice per group).

Mice were found to tolerate a single intraperitoneal dose of vitamin C (5 g/kg), which was comparable to that (4 g/kg) used in a previously published study (6). No obvious toxicity was observed although following drug administration, cooling of mouse skin and reduced motor activity was observed for several hours. Plasma concentrations of 5-HIAA rose significantly (*p* < 0.05 at 2 h; < 0.001 at 2 and 4 h) with increasing time in groups of three non-tumor-bearing mice treated with a single dose of vitamin C (5 g/kg), as shown in Figure 1A. The dose response of plasma 5-HIAA in groups of six non-tumor-bearing mice was also measured 2 h after a single dose of Vitamin C (Figure 1B); plasma concentrations increased significantly (*p* < 0.05) following doses of 2 and 4 g/kg. The plasma 5-HIAA response to vitamin C was compared to those of two established vascular disrupting agents. New data for groups of three non-tumor-bearing mice treated with fosbretabulin (25 mg/kg) are shown in Figure 2 and indicate that plasma 5-HIAA concentrations increase significantly (*p* < 0.05) at both time points. The results for fosbretabulin have been compared with previously published data (36) for non-tumor-bearing mice treated with vadimezan (25 mg/kg).

**Figure 3:**
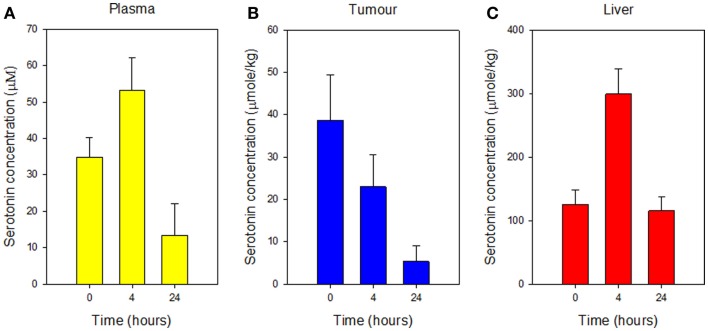
**Concentrations of serotonin in plasma (A), Colon 38 subcutaneous tumor tissue (B), and liver tissue (C), measured before or at the indicated time after i.p. administration of vitamin C (4 g/kg)**. Vertical bars represent SEM (three mice per group).

5-HIAA is a hepatic metabolite of serotonin, which is stored in platelets within tissues. Serotonin concentrations were therefore measured in plasma, tumor tissue, and liver tissue of groups of 3 Colon 38 tumor-bearing mice either 4 or 24 h after administration of a single dose of vitamin C (4 g/kg). Serotonin concentrations increased in plasma after 4 h, but the difference was not significant (Figure 3A). Serotonin concentrations in tumor tissue decreased significantly (*p* < 0.05) after 24 h (Figure 3B) but increased significantly (*p* < 0.05) in liver tissue after 4 h (Figure 3C).

### Effects of vitamin C treatment on Colon 38 tumor growth *in vivo*

**Figure 4:**
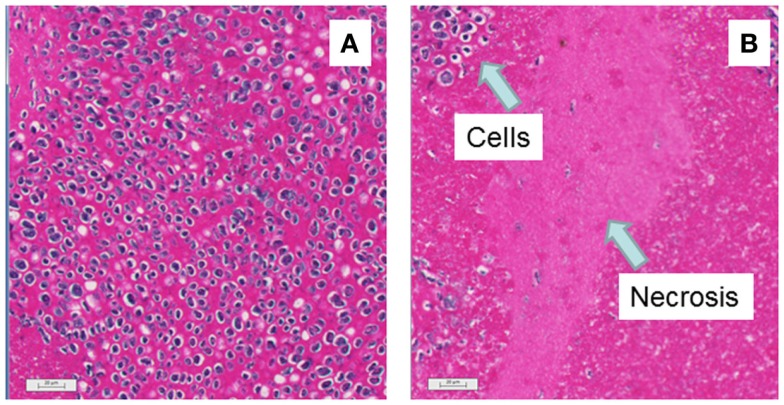
**Histological appearance of representative sections of mice with subcutaneous Colon 38 tumors is shown**. Bars indicate 20 μm. **(A)** No treatment: intact cells can be seen by purple nuclei with occasional pink spaces indicating necrosis. **(B)** Twenty-four hours after i.p. administration of vitamin C (4 g/kg): the large rather featureless pink regions indicate areas of hemorrhagic necrosis.

**Figure 5:**
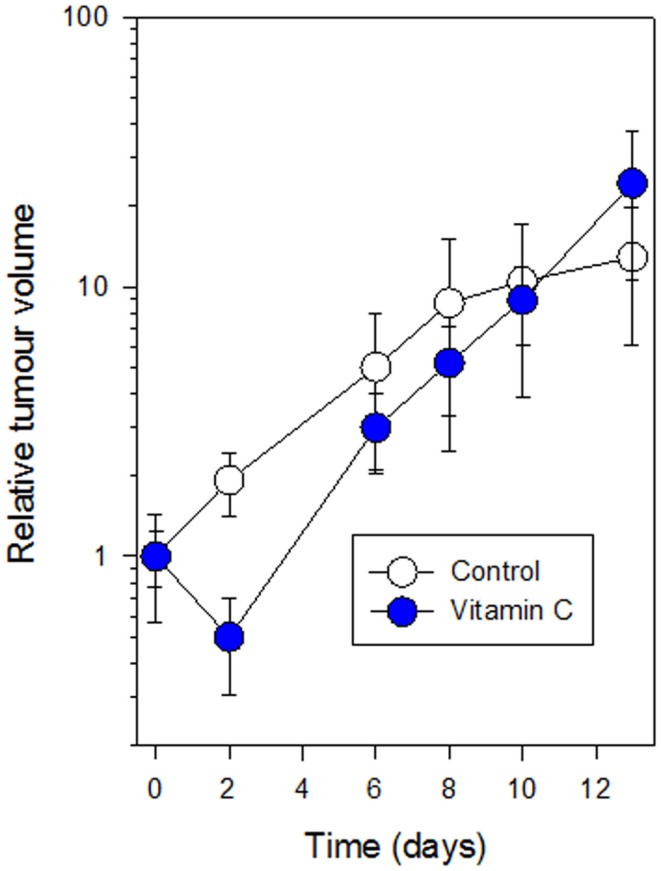
**Growth of subcutaneous Colon 38 tumors following i.p. administration of vitamin C (4 g/kg) (filled circles) as compared to control mice (open circles) is shown**. Vertical bars represent SEM (five mice per group).

Mice with subcutaneous Colon 38 murine adenocarcinomas were treated with a single dose of vitamin C (4 g/kg), and tumors were removed after 24 h for histology. Tumors from five vitamin C-treated mice were red in color while those from seven control mice were white. Stained tumor sections (Figure 4) from vitamin C-treated mice showed larger areas of necrotic cells (62 ± 4%) than did control mice (22 ± 5%) and the difference was significant (*p* < 0.01). Mice with subcutaneous Colon 38 murine adenocarcinomas (five per group; approximately 5 mm in diameter; 62 ± 16 mm^3^) were treated with a single dose of vitamin C (4 g/kg), and tumor volumes were measured at various times later. After 2 days, the tumor volumes (relative to the starting volume) were 1.92 ± 0.51 for the control group and 0.50 ± 0.19 for the treated group, a significant reduction (*p* = 0.03) than those of the control group, but thereafter tumor growth in the treated group was slightly higher than that in the control group and by 10 days, treated and control tumors had similar average volumes (Figure 5).

## Discussion

The results provide preliminary evidence for the hypothesis that high-dose vitamin C has a tumor vascular disrupting effect in mice. Tumors removed from mice 24 h after treatment with vitamin C were red in color while tumors from control mice were white; this was similar to what was found previously with vadimezan and other vascular disrupting agents (results not shown). Tumor sections from vitamin C-treated mice (Figure 4) showed significantly larger areas of necrotic tumor tissue than did those from control tumors (62 versus 22%, respectively), although the effect was not as great as that induced by vadimezan and several other vascular disrupting drugs. Tumor-bearing mice treated with vitamin C (4 g/kg) responded with an initial decrease in tumor volume after 2 days followed by a phase of more rapid tumor growth (Figure 5), again similar to that caused by a suboptimal dose of vadimezan. It is clear, at least with the Colon 38 tumor, that vitamin C administered at a single high dose is less effective than vadimezan, but it should be kept in mind that vadimezan may differ from vitamin C by having two actions, a direct one on tumor endothelial cells (37) and an indirect one mediated by cytokine induction (16, 38). Further experiments with multiple doses and other tumor types would be required to fully characterize this vascular disrupting effect of vitamin C.

Vascular disrupting agents affect the normal vascular endothelium as well as the tumor vascular endothelium (24). The increased plasma 5-HIAA concentrations in non-tumor-bearing mice in response to vitamin C (Figure 1) are consistent with an effect on normal vasculature. Cooling of mouse skin, observed for several hours after administration of the maximum tolerated dose of vitamin C (5 g/kg), resemble that observed in mice treated with vadimezan and also suggest an effect on normal endothelium. Skin cooling has been interpreted as arising from inhibition of capillary blood flow in normal skin (32). It was of interest that serotonin concentrations in liver were, in contrast to those in tumor tissue, elevated after 4 h (Figure 3C). The observation again suggests an effect on normal tissues and several explanations are possible, such as increased trafficking of platelets to the liver.

Vascular disrupting agents can be divided into two classes, the first comprising tubulin binders and the second comprising FAA, xanthenone acetic acid (XAA), and a variety of XAA analogs including vadimezan (16, 39). The present study suggests that vitamin C is a member of the second class, raising the question of whether acidic character is important for the activity of this class. It is clear at least from studies of FAA and XAA analogs in mice, that activity has quite exacting structural requirements (40). Cells normally exclude acidic molecules and uptake is likely to depend on the expression of appropriate drug transporters (41). In the case of vitamin C, uptake is thought to be strongly dependent on the expression of cellular vitamin C transporters; the SVCT2 transporter is highly expressed in both endothelial cells and macrophages and results in millimolar intracellular concentrations (2, 42). It would be interesting to determine whether the vascular disrupting activity of other members of this second class is dependent on the expression of transporters that are expressed in endothelial cells.

The molecular targets of vitamin C in endothelial cells are still being characterized. One possible target is tetrahydrobiopterin, whose stabilization by vitamin C leads to nitric oxide production, S-nitrosation of multiple proteins (43, 44), and increased endothelial permeability (45). Tetrahydrobiopterin is also an essential cofactor for tryptophan hydroxylase (46), and stabilization might also lead to increased serotonin production, potentially explaining the increased liver serotonin concentrations (Figure 3C). Another possible target of vitamin C is HIF-1 hydroxylase; by stabilizing this enzyme, vitamin C may inhibit induction of the transcription factor (HIF-1) and thus inhibit the induction of multiple genes by HIF-1 (47).

It is not yet possible to determine whether the effects observed here in mice are relevant to any effects of high-dose intravenous vitamin C in humans. Plasma concentrations of vitamin C were not measured in this study but published data with mice show that they are likely to reach 40 mM following a dose of 4 g/kg (6). Studies in humans administered high-dose intravenous vitamin C have reported comparable plasma concentrations of 24 mM (5). It seems likely that vascular disruptive properties of high-dose vitamin C, if they occur in human tumors, would not be the sole contributor to any possible antitumor effect; other cells that express SVCT2 transporters, such as cells of the innate immune system, might also be affected. In any future clinical trials designed to test the clinical effects of high-dose vitamin C administration, plasma 5-HIAA might prove to be a useful biomarker.

## Conflict of Interest Statement

The authors declare that the research was conducted in the absence of any commercial or financial relationships that could be construed as a potential conflict of interest.
